# The sacral networks and neural pathways used to elicit lumbar motor rhythm in the rodent spinal cord

**DOI:** 10.3389/fncir.2014.00143

**Published:** 2014-12-03

**Authors:** Meir Cherniak, Alex Etlin, Ido Strauss, Lili Anglister, Aharon Lev-Tov

**Affiliations:** Department of Medical Neurobiology, Institute for Medical Research—Israel-Canada, IMRIC, The Hebrew University Medical SchoolJerusalem, Israel

**Keywords:** sacrocaudal afferents, calcium imaging, spinal interneurons, ascending pathways, central pattern generators, locomotor, alpha1 adrenoceptors

## Abstract

Identification of neural networks and pathways involved in activation and modulation of spinal central pattern generators (CPGs) in the absence of the descending control from the brain is important for further understanding of neural control of movement and for developing innovative therapeutic approaches to improve the mobility of spinal cord injury patients. Activation of the hindlimb innervating segments by sacrocaudal (SC) afferent input and by specific application of neurochemicals to the sacral networks is feasible in the isolated spinal cord preparation of the newborn rat. Here we review our recent studies of sacral relay neurons with lumbar projections and evaluate their role in linking the sacral and thoracolumbar (TL) networks during different motor behaviors. Our major findings show that: (1) heterogeneous groups of dorsal, intermediate and ventral sacral-neurons with ventral and lateral ascending funicular projections mediate the activation of the locomotor CPGs through sacral sensory input; and (2) rhythmic excitation of lumbar flexor motoneurons, produced by bath application of alpha-1 adrenoceptor agonists to the sacral segments is mediated exclusively by ventral clusters of sacral-neurons with lumbar projections through the ventral funiculus.

## Introduction: the thoracolumbar limb-moving and sacrocaudal body-stabilizing rhythmogenic networks are tightly coupled

**Figure 1:**
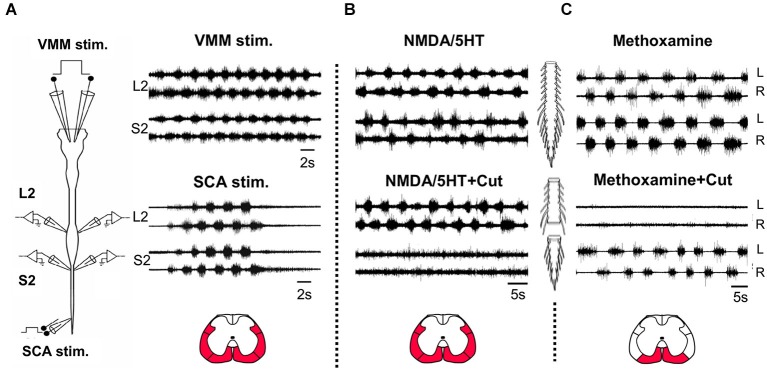
**Coordinated activation of the lumbar and sacral rhythmogenic networks. (A)** Schematic illustration of the isolated brainstem spinal cord preparation of the neonatal rat (left). Stimulation electrodes are bilaterally attached to the ventromedial medulla (VMM) and to sacrocaudal afferents (SCA) entering though S4 or Co1 dorsal roots. Signals are recorded from left and right (L and R) ventral roots of L2 and S2. Right: Recordings of the rhythm produced by VMM (top) and SCA (bottom) stimulation from the left and right L2 and S2 ventral roots. VMM stimulation: 5 ms, 80-pulse 4 Hz train at 1 mA; SCA stimulation: 0.2 ms, 50-pulse 4 Hz train at 25 μA for the SCA. **(B)** The rhythm produced by bath application of 4 μM NMDA and 10 μM 5HT in the isolated spinal cord preparation of the newborn rat and recorded from the left and right L2 and S2 ventral roots, before (NMDA/5HT) and after transection of the cord through the lumbosacral junction (NMDA+5HT+Cut). **(C)** The rhythm produced by bath application of methoxamine (100 μM) in the isolated spinal cord preparation of the newborn rat and recorded from the left and right L2 and S2 ventral roots, before (Methoxamine) and after (Methoxamine+Cut) transection of the cord through the lumbosacral junction. The red regions in the cross sections illustrated below denote the major white matter funiculi at the lumbosacral junction, linking the sacral and lumbar networks to produce coordinated rhythmic activity under the experimental conditions specified in (**A**), (**B**) and (**C**), respectively.

Spinal neuronal networks known as central pattern generators (CPGs) produce the rhythmic patterned output required for coordinated movements such as swimming and stepping in many species of vertebrates including humans (for reviews see Alford et al., 2003; Kiehn, 2006; Hultborn and Nielsen, 2007; Frigon, 2012). The CPGs are controlled by descending supraspinal commands and can be modulated to produce different patterns and behaviors. However, the CPGs can be also activated and modulated in the absence of descending control from brain by afferent-input and neuroactive compounds. Indeed, recent clinical studies have shown that reactivation of the CPGs in spinal cord injury patients by afferent input is possible and that it improves the motor function and mobility of some of the patients (for review see Hubli and Dietz, 2013). Therefore, it is of particular interest and significance to elucidate the networks and pathways involved in activation and modulation of the CPGs in accessible experimental models. The pattern generating circuitry in rodents activates the limb, trunk, and axial muscles innervating-segments to produce stabilized and coordinated locomotion under different conditions. The rhythmogenic capacity of these networks is preserved *in vitro*, in the isolated spinal cord preparation of newborn rodents (e.g., Kudo and Yamada, 1987; Smith et al., 1988). Therefore, this preparation can be used as an ideal model to study the mechanisms and interactions of the body stabilizing and limb moving networks in the absence of supraspinal control. In previous studies we described a rhythmogenic network in the sacrocaudal (SC) segments of the neonatal rat spinal cord that controls the axial and tail musculature during various movements (Lev-Tov and Delvolvé, 2000; Lev-Tov et al., 2000; Delvolvé et al., 2001; Gabbay et al., 2002). The SC-rhythmogenic network could be activated in surgically detached sacral spinal segments by stimulation of sacrocaudal afferents (SCA; Lev-Tov et al., 2000; Delvolvé et al., 2001), by bath application of noradrenaline (NA) and NMDA (Gabbay and Lev-Tov, 2004) and by the alpha1-adrenoceptor agonist, methoxamine (Gabbay and Lev-Tov, 2004). Figure 1 shows that the SC rhythmogenic network is co-activated with the hindlimb CPGs in the isolated spinal cord of the neonatal rat in the absence and presence of bath-applied neurochemicals. Coordinated activation of the SC- and locomotor-CPGs could be also demonstrated in response to electrical stimulation of the ventromedial medulla (VMM; Blivis et al., 2007; e.g., Zaporozhets et al., 2004) or SCA in the isolated brainstem-spinal cord preparation (Figure 1A, see Blivis et al., 2007; e.g., Gordon et al., 2008).

Most of the studies of rodent rhythmogenesis used various combinations of NMDA and monoamines to produce the rhythm (reviewed in Miles and Sillar, 2011). Figure 1B shows that the NMDA/5HT induced rhythm in the sacral spinal segments is nearly abolished when the spinal cord is transected at the lumbosacral junction, while the 0.25–1 Hz lumbar rhythm produced in the presence of methoxamine disappears when the sacral segments are disconnected from the lumbar spinal cord (Figure 1C). These findings suggest that the lumbar-CPGs drive the sacral-CPGs in the presence of NMDA/5HT and that the sacral-CPGs drive the rostral-lumbar segments to produce rhythmic bursting in the presence of methoxamine (see also Gabbay and Lev-Tov, 2004). The coupling between the thoracolumbar (TL) and SC-CPGs during the rhythm induced by sensory and brainstem stimulation was found to be in the rostro-caudal direction (Bonnot et al., 2002; Lev-Tov and O’Donovan, 2009; Etlin et al., 2010; Lev-Tov et al., 2010; Strauss, 2011). On the other hand, caudo-rostral coupling between spinal networks is also well known in many vertebrates (see changes in the direction of propagation when switching from forward to backwards swimming in lampreys, Islam et al., 2006) including the neonatal rat spinal cord preparation during neurochemically induced motor rhythms (Falgairolle and Cazalets, 2007).

Our interest in the SC-network stems from two main reasons. First, it provides basic knowledge regarding stabilization of the body axis and the use of the tail musculature during rhythmic limb movements. Second, SC-interneurons may serve as important relays onto the locomotor generators during a nocifensive/escape type of behavior triggered by stimulation of SC dermatomes (Smith et al., 1988; Lev-Tov and Delvolvé, 2000; Lev-Tov et al., 2000; Whelan et al., 2000; Delvolvé et al., 2001; Bonnot et al., 2002; Strauss and Lev-Tov, 2003). Moreover, the ability to activate the locomotor CPGs in the absence of descending control from the brain via sacral relays has a potential clinical significance for future treatments of spinal cord injury patients (see below).

More specifically, the capacity to produce the locomotor rhythm by SCA depends largely on synaptic activation of sacral relay neurons with lumbar projections (Strauss and Lev-Tov, 2003; Blivis et al., 2007; Etlin et al., 2010, 2013; Lev-Tov et al., 2010). The ability of sacral CPGs to produce an alternating left-right lumbar-flexor bursting in response to sacral application of methoxamine, also depends on activation of sacral relay neurons with rostral lumbar projections (Gabbay and Lev-Tov, 2004; as in Figure 1C). Here we briefly review our studies of the mediating relay neurons and the pathways involved in linking the sacral and lumbar networks under these conditions and discuss possible mechanisms and implications of our findings.

## I- Activation of locomotor CPGs by sacrocaudal afferent input

### Mediation of SCA-induced locomotor activity by sacral relay-neurons with lumbar projections

Afferent input is a potent activator of the locomotor CPGs in the absence of the descending control from the brain in humans and experimental mammalian models (Grillner and Rossignol, 1978; Grillner and Zangger, 1979; Duysens and Pearson, 1980; Pearson and Rossignol, 1991; Prochazka et al., 1997; Dietz and Duysens, 2000; Dietz et al., 2002; Pearson, 2004; for reviews see Edgerton et al., 2008; Dietz, 2009). We showed that mechanical and radiant heat stimulation of SC dermatomes (Lev-Tov et al., 2000; and Blivis et al., 2007, respectively; e.g., Mandadi and Whelan, 2009) as well as electrical stimulation of SCA induces coordinated activation of the locomotor and SC-CPGs (Lev-Tov et al., 2000; Strauss and Lev-Tov, 2003; Klein and Tresch, 2010; for the mouse see Whelan et al., 2000).

The SCA-induced locomotor rhythm in the newborn rat spinal cord, could be blocked by bathing the sacral segments in low-calcium high-magnesium Kreb’s saline, by selective sacral application of the non-NMDA receptor blocker CNQX (Strauss and Lev-Tov, 2003) or the mu-opioid agonist DAMGO (Blivis et al., 2007). Using surgical and pharmacological manipulations of the spinal cord, we found that the sacral relay neurons, activated by SCA to produce the locomotor rhythm, project to the lumbar segments through the ventral (VF), ventrolateral/lateral (VLF/LF) and dorsolateral (DLF) white matter funiculi (Strauss and Lev-Tov, 2003; Etlin et al., 2010; Lev-Tov et al., 2010). These studies further showed that the capacity of SCA input to produce the locomotor rhythm depends not only on activation of sacral relay-neurons with direct projections to the lumbar CPGs, but also on serial recruitment of multi-funicular sacral-propriospinal neurons interposed between the second order neurons and the hindlimb-CPGs.

### Segmental and spatial distribution of sacral relay neurons with lumbar projections

**Figure 2:**
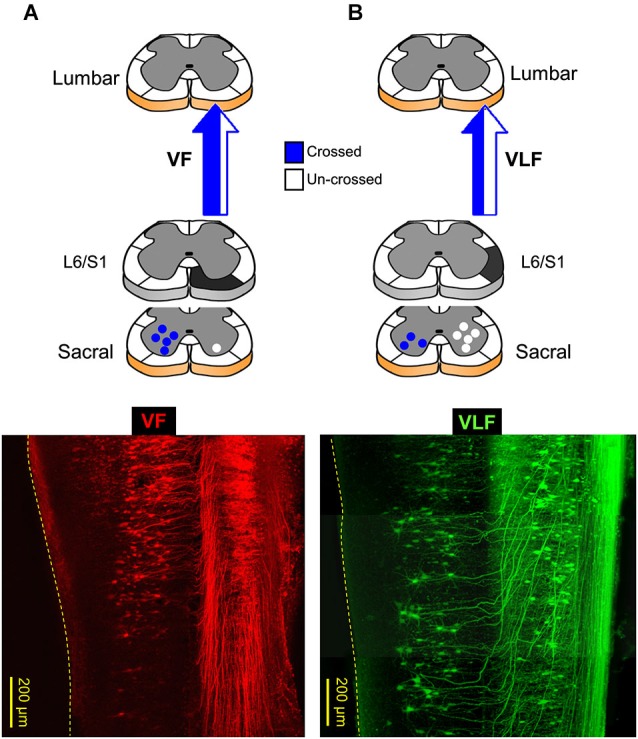
**Lumbar projections of sacral relay neurons through the ventral and ventrolateral white matter funiculi (VF and VLF; A and B, respectively)**. Bottom panels are confocal projected images of two transparent whole-mount spinal cord preparations in which sacral neurons with lumbar projections were back-labeled through the VF (**A**, red) and VLF (**B**, green). On top are illustrations of lumbar projections through the VF and VLF. **(A)** The lumbar projections of sacral neurons, labeled through the VF at the lumbosacral junction (L6/S1, black), are mainly crossed (blue). **(B)** More than 60% of the lumbar projections of sacral neurons, labeled through the VLF at the lumbosacral junction (L6/S1, black), are un-crossed (white). Note that the crossed lumbar projections enter the VLF after traveling along the VF (VLF micrograph; e.g., Etlin et al., 2010). The projections through the VLF are the combined projections through VLF/LF as originally described in Etlin et al. (2010).

Confocal imaging of sacral relay neurons with lumbar projections, back-labeled through cut VF, VLF/LF and DLF axon bundles at the lumbosacral junction, was used to map their segmental and spatial distribution, and lumbar projection patterns (Etlin et al., 2010; Lev-Tov et al., 2010). Figure 2 shows low-power projected confocal images of sacral relay-neurons in whole-mount transparent preparations of the spinal cord, labeled through the cut VF (Figure 2A) and the combined VLF/LF (“VLF”, Figure 2B) axons at the left lumbosacral junction (see, respective scheme above each micrograph; also Etlin et al., 2010). These confocal micrographs demonstrate that most of the lumbar VF projections are crossed, and the majority of the lumbar VLF/LF projections are un-crossed (see also the schemes in A and B: crossed projections in blue, uncrossed projections in white). The DLF lumbar projections (not shown) were uncrossed. The back-labeled DLF-neurons were revealed mainly within the dorsal horn laminae, while most of the VF- and VLF/LF-neurons were in laminae VII, VIII and IX and the deep dorsal horn laminae (Etlin et al., 2010).

### Sacrocaudal primary afferents and intraspinal neurons innervate sacral relay-neurons with lumbar projections

After mapping the distribution of sacral relay-neurons and their lumbar projection patterns, we examined whether these neurons are contacted by SCA, and whether these contacts are glutamatergic as predicted by Strauss and Lev-Tov (2003). Using back-labeling of sacral VF-neurons, anterograde labeling of SCA and immunostaining for vesicular glutamate transporters 1 and 2, we found that many VF-neurons are innervated by primary afferents immunoreactive for VGluT1, some VF-neurons were innervated by VGluT2^+^ primary afferents, and large proportions of the VF-neurons received VGluT2^+^ contacts from intraspinal neurons (Etlin et al., 2013). These findings suggest (e.g., Alvarez et al., 2004; Liu et al., 2010; Scherrer et al., 2010; Rogoz et al., 2012) that the sacral VF-neurons can be activated directly by glutamatergic proprioceptive and nociceptive primary afferents and indirectly by glutamatergic-interneurons (Etlin et al., 2013).

### Sacral VF-neurons serve as an important link between SCA and the hindlimb-CPGs

Etlin et al. (2010) have shown that bilateral interruption of the VF at the lumbosacral junction caused more severe interference to the lumbar rhythm produced by SCA stimulation than lesion of any other pair of white matter funiculi. They also showed that following multifunicular lesions that spared only one of the funiculi, the most regular and robust lumbar rhythm was obtained when the VF was left bilaterally intact (Etlin et al., 2010). Therefore, sacral relay-neurons projecting to the lumbar segments through the VF (VF-neurons) have been suggested to contribute more substantially than other sacral relay-neurons to activation of the lumbar-CPGs (Strauss and Lev-Tov, 2003; Etlin et al., 2010; Lev-Tov et al., 2010). These VF-neurons are innervated by glutamatergic SCA and intraspinal neurons (see above). The question raised next is whether VF-neurons are activated by SCA-stimulation, and if so, how their activity is related to the concomitant motor-output.

**Figure 3:**
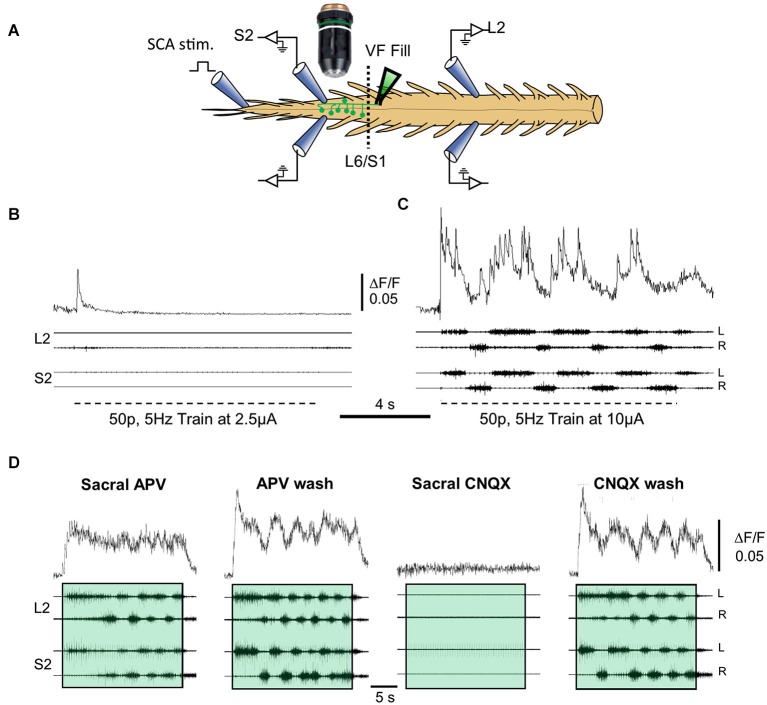
**Correlation between the activity of VF neurons and the motor output during SCA stimulation. (A)** Illustration of the experimental set up used for simultaneous imaging of VF neurons and electrophysiological recordings of the motor output during the rhythm produced by SCA stimulation. Sacral relay neurons are loaded with calcium green dextran through the cut VF at the lumbosacral junction (L6/S1) in the isolated spinal cord preparation of the neonatal rat (VF fill). The preparation is mounted with ventral side up (in this example) in a dual chamber experimental bath (dashed line at the L6/S1 denotes the Vaseline barrier separating the two chambers). The motor output produced by stimulation of Co1 or S4 dorsal roots (SCA stimulation) is recorded from the left and right L2 and S2 ventral roots. Calcium transients produced in fluorescently labeled sacral VF-neurons are imaged simultaneously using epifluorescence microscopy with attached 14 bit CCD camera (for further details see Etlin et al., 2013). The difference image (ΔF/F) of imaged VF neurons and the simultaneous L2 and S2 ventral root recordings during SCA stimulation under different conditions are superimposed in **(B–D)**. The relations between the optical and electrophysiological signals are analyzed using the cross Wavelet and cross Wavelet coherence methods (Mor and Lev-Tov, 2007; Etlin et al., 2013). **(B,C)** Top traces are the activity patterns imaged from a left S2 VF-neuron (ΔF/F) back-labeled from the right VF with Calcium green dextran during 0.1 ms, 50-pulse, 5 Hz stimulus trains applied to the Co1 dorsal root at 2.5 and 10 μA (**B** and **C**, respectively). Below are the corresponding motor outputs (four bottom traces) recorded from the left and right ventral roots of S2 and L2, simultaneously with the optical signals. **(D)** The activity imaged from a VF-neuron (ΔF/F, upper records) and the motor output recorded from the left and right S2 and L2 ventral roots (four bottom records), during stimulation of the Co1 dorsal root, in the presence of 20 μM of sacrally applied APV (Sacral APV) and 35 min after APV wash (APV wash). Sacral addition of 10 μM CNQX under these conditions (Sacral CNQX) blocked the activity. The block is alleviated 60 min after CNQX wash (CNQX wash). Trains are confined within colored transparent rectangles. The oscillatory drive of the cells is markedly reduced by APV and is abolished by CNQX. For further details, see text and Etlin et al. (2013). Fifty-pulse 2.5 Hz stimulus trains were applied at 10 μA to produce the rhythm. **(B–D)** Modified from Etlin et al. (2013).

Figure 3 demonstrates calcium imaging of back-filled VF-neurons and concurrent electrophysiological recordings of the motor-output produced in the rostral-lumbar and sacral segments of the isolated spinal cord during SCA stimulation (Figure 3A). Recordings were obtained from the ventral surface of the cord after prolonged back-loading with the calcium sensor (Calcium green dextran) through cut bundles of right VF-axons at the lumbosacral junction. The fluorescence changes (ΔF/F) produced by calcium transients during the stimulus trains applied to SCA at two different intensities are shown in Figures 3B,C. The rhythmic ΔF/F of the imaged left VF-neuron during the higher-intensity stimulus train, exhibits a characteristic rhythmic pattern, in phase with the left ventral root bursting (Figure 3C). Our experiments revealed that more than 50% of the back-labeled VF-neurons could be activated by SCA stimulation. Forty-one percent of the activated VF-neurons exhibited tonic pattern, 18% showed pure rhythmic pattern, 34.5% had a tonic pattern with superimposed oscillations, and 6.2% fired irregularly during the stimulus trains. Analysis of the 161 crossed-projecting VF-neurons that exhibited oscillatory drive (with and without background tonic activity), revealed that ~70% of them were in phase with the ipsilateral-motor output and ~30% were in phase with the contralateral motor output (Etlin et al., 2013). We have suggested that VF-neurons with ipsilateral phase-preference are crossed-inhibitory and those with contralateral phase-preference are crossed-excitatory commissural neurons with lumbar projections (Etlin et al., 2013). This suggestion is based on the special pattern produced by sacral networks (alternating left-right rhythm and in-phase flexor-extensor activation at a given side of the sacral cord), and on studies of the phase preference of Vo commissural neurons (Lanuza et al., 2004) and lamina VIII GABAergic neurons (Wu et al., 2011).

Another interesting finding involves the relation between rhythmic bursting of the sacral CPG, the activity pattern of VF-neurons, and lumbar motor-output. Figure 3D demonstrates that application of the NMDA-receptor blocker APV to the sacral segments reduces the activity of the sacral-CPGs without blocking the concomitant lumbar-rhythm. Under these conditions, the tonic drive of the imaged VF-neurons is not altered (compare Sacral APV to APV wash) while the oscillatory drive of the neurons is completely blocked in ~60% of the cells with the combined rhythmic/tonic pattern and of the cells with the rhythmic pattern, and is markedly attenuated in the remaining ~40% of these cells (Etlin et al., 2013). When APV is washed out, the sacral-CPGs are vigorously activated; a strong rhythmic drive develops in the imaged neuron and the concomitant lumbar motor-output is strengthened significantly. All optical and electrophysiological activities are abolished in the presence of the non-NMDA receptor antagonist CNQX (Sacral CNQX), and reappear after washing the CNQX (CNQX wash). Collectively these findings suggest the following: (1) the ability of SCA stimulation to activate the VF-neurons and the CPGs depends on non-NMDA receptor-mediated synaptic transmission in the sacral segments; (2) activation of the lumbar CPGs by SCA stimulation can be obtained when the activity of the sacral CPGs is reduced or nearly blocked (see also Strauss and Lev-Tov, 2003), and when the drive of the recruited VF-neurons is purely tonic; and (3) the activities of VF-neurons and the locomotor CPGs are maximized when the SC CPGs are activated. Thus, sacral VF-neurons are suggested to be a significant link between SCA and the locomotor CPGs.

## II. Caudo-rostral coupling between sacral and rostral lumbar networks during alpha1-adrenoceptor agonist activation of the sacral CPGs

**Figure 4:**
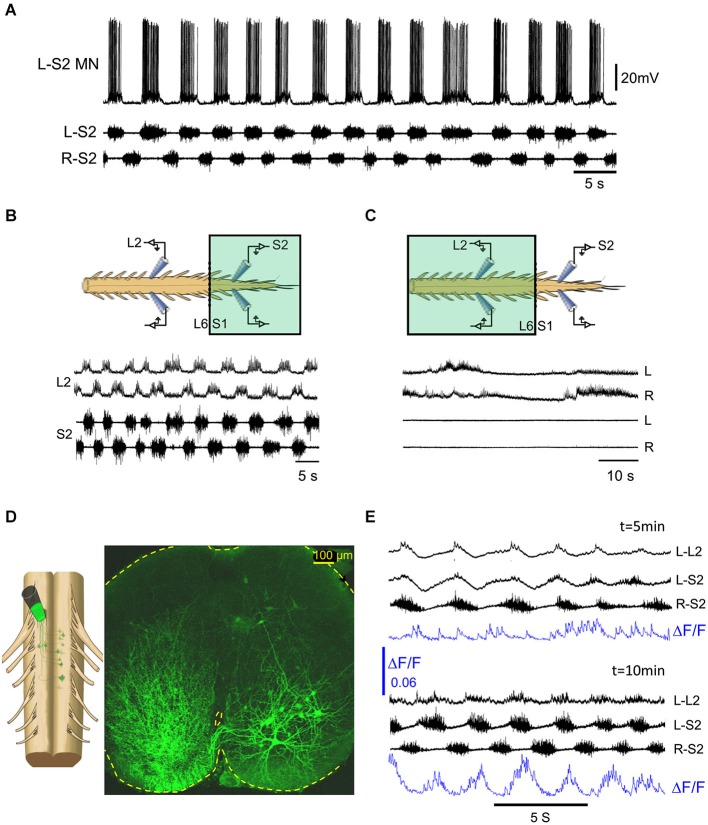
**The methoxamine-induced 0.25–1 Hz rhythm in the sacral spinal segments is relayed to the rostral lumbar network by sub-clusters of ventrally located sacral VF neurons. (A)** Intracellular recordings from a left S2 motoneuron (L-S2 MN) are superimposed with concurrent recordings from the left and right S2 ventral roots in the presence of bath-applied methoxamine (100 μM) to the surgically detached sacral segments of the spinal cord. **(B)** Recordings from the left and right L2 and S2 ventral roots showing alternating left-right bursting when methoxamine is applied to the sacral compartment (colored rectangle) of a dual chamber experimental bath. **(C)** Recordings from the left and right L2 and S2 ventral roots in the experiment described in **(B)** after washing the sacrally applied methoxamine and adding methoxamine to the TL compartment (colored rectangle) of the experimental bath. The sacral rhythmic activity is blocked, while a slow rhythm appears in the lumbar segments. **(D)** Confocal micrograph of a 70 μm cross section through the S2 spinal segment shows left sacral neurons back-loaded with fluorescein-dextran through the right VF at the lumbosacral junction (dye back-loading is illustrated on the left). **(E)** Imaging the activity developed in a ventral S2 VF-neuron back-loaded with Calcium green dextran and viewed from the ventral aspect of the isolated spinal cord (blue, ΔF/F) and the concurrently recorded motor output from the left L2 and the left and right S2 ventral roots, 5 and 10 min after addition of methoxamine to the experimental bath. The 0.1 Hz–10 Khz ventral root recordings were not high-pass filtered to reveal the early subthreshold activity in L–L2.

The spinal cord receives extensive noradrenergic innervation (e.g., Gabbay and Lev-Tov, 2004). Noradrenaline is known to initiate locomotor activity and modulate locomotor-like activity in a number of adult mammals (For review see Miles and Sillar, 2011). In the isolated spinal cord of neonatal rodents, NA produces a very slow rhythm with various irregularities (Kiehn et al., 1999; Cazalets and Bertrand, 2000; Sqalli-Houssaini and Cazalets, 2000). When applied to the sacral segments of the neonatal rat spinal cord, NA produces a short lasting (1–2 min) “fast” alternating left-right rhythm in the sacral and lumbar segments before transforming into a very slow non-locomotor rhythm (Gabbay and Lev-Tov, 2004). The “fast” NA rhythm was found to be blocked by alpha1 and not by alpha2 adrenoceptor antagonists (Gabbay et al., 2002). A “fast” (0.25–1 Hz) and robust alternating left-right rhythm could be produced and maintained in the isolated spinal cord of neonatal rats, in the presence of the alpha1-adrenoceptor agonist methoxamine (see Figures 1, 4). Our findings, that the methoxamine rhythm persists in the sacral segments and is blocked in the lumbar segments after transecting the spinal cord at the lumbosacral junction (Figure 1C, and Gabbay and Lev-Tov, 2004), suggest that the rhythm originates in the sacral segments of the spinal cord. The experiment shown in Figures 4B,C demonstrates that the 0.25–1 Hz methoxamine rhythm appears in both sacral and lumbar segments of the spinal cord only when methoxamine is added to the sacral but not to the TL spinal segments. Thus, there must be a potent coupling between the sacral CPGs and the rostral lumbar spinal segments.

What is the nature of the lumbar bursting produced by sacrally applied methoxamine? Gabbay and Lev-Tov (2004) showed that addition of methoxamine to the sacral segments produces rhythmic bursts in lumbar flexor but not in extensor motoneurons. They provided evidence suggesting that the sacral-CPGs do not activate the lumbar-CPGs in the presence of sacral methoxamine, but rather activate lumbar flexor-motoneurons and lumbar commissural-neurons (see Figure 9 in Gabbay and Lev-Tov, 2004, for the hypothetical organization of the circuitry). To study the link between the sacral-CPGs and the rostral lumbar segments under these conditions, we tried to determine the minimal anatomical configuration that is required to produce the 0.25–1 Hz rhythm in rostral lumbar motoneurons by sacrally applied methoxamine. Our most recent data (see abstracts by Cherniak et al., 2011; Roisman et al., 2014), revealed that: (1) the ability of methoxamine to produce the 0.25–1 Hz lumbar rhythm depends on intact connectivity between the TL and the first two sacral spinal segments (S1–S2); (2) the lumbar rhythm persists after removal of the dorsal aspect of the sacral cord down to the central canal, but not below it; and (3) the methoxamine-induced lumbar rhythm depends on ventral sacral-neurons projecting rostrally only through ventral funiculi. Following the latter finding, we imaged the activity of ventral clusters of back-labeled VF-neurons in the presence of methoxamine (Figure 4D) and studied their relation to the concomitant motor-output produced in the sacral- and lumbar-segments. The activity pattern of these ventral clusters of sacral VF-neurons in the presence of methoxamine was rhythmic in most cases with phase preference mainly to the ipsilateral motor output. Figure 4E shows the gradual appearance of rhythmic calcium transients in a left VF-neuron back-loaded with calcium green dextran and rhythmic bursting in sacral- and lumbar-segments in the presence of methoxamine. Regular optical and electrophysiological oscillatory signals developed after 10 min exposure to methoxamine. The ΔF/F of the left VF neuron imaged in this experiment is in phase with the ipsilateral motor output (L-S2).

We suggest that sub-clusters of ventrally located sacral-neurons mediate the generation of the rhythmic bursting in lumbar flexor-motoneurons by sacrally applied methoxamine. Studies of trans-synaptic labeling of sacral VF- and other sacral-interneurons, using GFP-encoded retrograde virus injection to hindlimb muscles (e.g., Hadas et al., 2014), suggest oligo-synaptic connectivity between sacral VF-neurons and rostral lumbar motoneurons. Additional experiments are needed to further clarify this issue and determine the functionality of this connectivity.

## Conclusions and suggestions

In this manuscript, we focused on the mediating role of sacral-neurons in linking the sacral and lumbar networks during rhythmic motor activity produced by SCA input and by selective activation of sacral-neurons by alpha1 adrenoceptor agonists. While SCA stimulation activates successfully both the sacral and lumbar CPGs, sacrally applied methoxamine activates the sacral CPGs and thereby produces alternating left-right bursting in rostral lumbar motoneurons. The lumbar rhythmic bursting produced by SCA stimulation is mediated mainly by relay-neurons with direct and indirect multifunicular lumbar-projections, while the methoxamine-induced rhythm is mediated by sacral-neurons that project rostrally only through the VF. We provided evidence that the drive produced by the activated VF relay neurons and the pathways associated with them, may turn-on the locomotor pattern generators when the sacral afferents are stimulated, and stimulate lumbar flexor motoneurons in the presence of methoxamine. Thus, sub-populations of sacral VF-neurons are suggested to project to flexor motoneurons that are driven by the more excitable and dominant rostral lumbar oscillators (e.g., Cazalets et al., 1995; Kjaerulff and Kiehn, 1996; Cowley and Schmidt, 1997; Kremer and Lev-Tov, 1997; Bonnot et al., 2002; for review see Kiehn, 2006), and to the locomotor CPGs (Etlin et al., 2010, 2013). Modulation of the activity of these two sets of projections enables separate and/or simultaneous control on the frequency and power of the rhythmic lumbar output under different conditions (see Finkel et al., 2014), and thereby plays a significant role in shaping the final motor-output.

Further studies are required to clarify the role of short- and long-ascending propriospinal pathways from the sacral to the lumbar cord (Bras et al., 1988; Grottel et al., 1998; Dutton et al., 2006), and of lumbar collaterals of spinothalamic, spinocerebellar or spinoreticular pathways (Leah et al., 1988; Edgley and Grant, 1991; Yamada et al., 1991; Katter et al., 1996; Matsushita, 1999; Garifoli et al., 2006) in activation of the locomotor CPGs, and the exact mechanisms by which inter-enlargements coupling is achieved. It is also important to verify whether it is possible to extrapolate our findings in newborn rodent to the adult spinal cord, and to evaluate the potential clinical significance of the ability to activate and modulate the CPG action via sacral relay neurons in the absence of descending supraspinal control.

## Conflict of interest statement

The authors declare that the research was conducted in the absence of any commercial or financial relationships that could be construed as a potential conflict of interest.
